# Tobacco Use Among Working Adults — United States, 2014–2016

**DOI:** 10.15585/mmwr.mm6642a2

**Published:** 2017-10-27

**Authors:** Girija Syamlal, Brian A. King, Jacek M. Mazurek

**Affiliations:** ^1^Respiratory Health Division, National Institute for Occupational Safety and Health, CDC; ^2^Office on Smoking and Health, National Center for Chronic Disease Prevention and Health Promotion, CDC.

Cigarette smoking has declined considerably among U.S. adults over several decades (*1*); however, increases have occurred in the use of noncigarette tobacco products in recent years, and the use of multiple tobacco products has become common among current users of noncigarette tobacco products (*2*,*3*). Differences in tobacco use have also been observed across population subgroups, including among working adults (*2*,*4*). CDC analyzed National Health Interview Survey (NHIS) data for 2014–2016 to describe the most recent prevalence estimates of current (every day or some days) tobacco product use among working U.S. adults by industry and occupation. Among working adults, 22.1% (32.7 million) currently used any form of tobacco; 15.4% used cigarettes, 5.8% used other combustible tobacco (cigars, pipes, water pipes or hookahs, very small cigars, and bidis), 3.0% used smokeless tobacco, and 3.6% used electronic cigarettes (e-cigarettes); 4.6% (6.9 million) reported current use of two or more tobacco products. By industry, any tobacco use ranged from 11.0% among education services to 34.3% among construction workers; current use of two or more tobacco products was highest among construction workers (7.1%). By occupation, any tobacco use ranged from 9.3% among life, physical, and social science workers to 37.2% among installation, maintenance, and repair workers; current use of two or more tobacco products was highest among installation, maintenance, and repair workers (10.1%). Proven interventions to prevent and reduce tobacco product use, including current use of multiple products, among working adults are important (*5*,*6*). Workplace tobacco-control interventions have been especially effective in reducing cigarette smoking prevalence (*7*).

NHIS data* are collected annually from a nationally representative sample of the noninstitutionalized U.S. population through a personal interview. Basic health and demographic information is collected for all family members. One adult aged ≥18 years per family is randomly selected to participate in the NHIS Sample Adult component of the survey, which contains questions on employment status and tobacco use. To improve the precision and reliability of estimates, NHIS data collected during 2014–2016 were combined. The NHIS Sample Adult component included 36,697 respondents in 2014, 33,672 respondents in 2015, and 33,028 respondents in 2016; response rates for those years were 60.8%, 55.2%, and 54.3%, respectively. The analysis was restricted to working adults (59,690; 57.7%). Respondents were considered to be currently working if, when asked about their employment status during the week before their interview, they reported that they were “working at a job or business,” “with a job or business but not at work,” or “working, but not for pay, at a family-owned job or business.” Information on participants’ current industry and occupation was coded by trained coders and grouped into 21 industry groups and 23 occupation groups.^†^

Current cigarette smokers were defined as respondents who reported having smoked ≥100 cigarettes during their lifetime and who reported now smoking “every day” or “some days.” Current other combustible tobacco smokers were those who reported smoking tobacco products other than cigarettes (including cigars, pipes, water pipes or hookahs, very small cigars, and bidis) at least once during their lifetime and currently smoking “every day” or “some days.” Current smokeless tobacco users were those who reported using smokeless tobacco products (including chewing tobacco, snuff, dip, snus, or dissolvable tobacco) at least once during their lifetime and who currently use “every day” or “some days.” Current e-cigarette users were those who reported using e-cigarettes at least once during their lifetime and current use “every day” or “some days.” Any current tobacco users were those who reported using one or more tobacco products (cigarettes, other combustible tobacco products, smokeless tobacco, or e-cigarettes). Multiple tobacco users were those who reported current use of two or more tobacco products.

Data were adjusted for nonresponse and weighted to be nationally representative. Prevalence estimates and corresponding 95% confidence intervals were calculated overall and by sociodemographic characteristics, industry, and occupation. Estimates with a relative standard error >30% are not reported. Two-sided t-tests^§^ were used to determine statistically significant (p<0.05) differences between point estimates.

During 2014–2016, among the annual estimated 242 million adults aged ≥18 years, 148 million (61.2%) were employed during the week before the interview. Among currently employed adults, 22.1% currently used any form of tobacco, including 15.4% who used cigarettes, 5.8% who used other combustible tobacco, 3.0% who used smokeless tobacco, and 3.6% who used e-cigarettes; 4.6% reported using two or more tobacco products.

Any current tobacco use was highest among men (27.4%), non-Hispanic whites (whites) (24.8%), persons aged 18–34 years (24.9%), those with high school education or less (30.1%), those with no health insurance (33.9%), those living below the federal poverty level^¶^ (28.5%), and those living in the Midwest (25.8%). Multiple tobacco product use was highest among men (6.5%), whites (5.5%), persons aged 18–34 years (6.0%), persons with a high school education or less (6.2%), and persons with no health insurance (7.7%) (Table 1).

**TABLE 1:** Estimated prevalence of current tobacco use among working* adults, by product type and selected characteristics — National Health Interview Survey, United States, 2014–2016

Characteristic	No. currently employed adults^†^ (x 1000)	% (95%CI)					
		Cigarette smokers^§^	Other combustible tobacco products^¶^	Smokeless tobacco products**	E-cigarettes^††^	Any tobacco product^§§^	≥2 Tobacco products^¶¶^
**Total (100%)**	**148,481**	**15.4 (15.0–15.8)**	**5.8 (5.5–6.1)**	**3.0 (2.8–3.3)**	**3.6 (3.3–3.8)**	**22.1 (21.6–22.6)**	**4.6 (4.4–4.9)**
**Age group (yrs)**							
≥18–34	51,289	16.3 (15.5–17.1)	7.9 (7.4–8.5)	3.6 (3.3–4.0)	4.8 (4.4–5.2)	24.8 (23.9–25.8)***	6.0 (5.6–6.5)***
≥35–54	64,600	16.2 (15.6–16.8)	5.0 (4.6–5.5)	3.2 (2.9–3.5)	3.5 (3.1–3.8)	22.6(21.9–22.3)	4.4 (4.1–4.8)
≥55	32,592	12.4 (11.7–13.1)	3.9 (3.4–4.3)	1.7 (1.4–2.0)	1.9 (1.6–2.2)	16.6 (15.8–17.4)	2.8 (2.4–3.1)
**Sex**							
Men	78,858	16.9 (16.3–17.5)	9.0 (8.6–9.5)	5.5 (5.1–5.9)	4.3 (3.9–4.6)	27.4 (26.7–28.2)***	6.5 (6.0–6.9)***
Women	69,623	13.7 (13.2–14.3)	2.1 (1.9–2.3)	0.2 (0.1–0.3)	2.8 (2.6–3.1)	16.0 (15.4–16.5)	2.6 (2.3–2.8)
**Race/Ethnicity**							
Hispanic	24,331	11.2 (10.3–12.1)	3.8 (3.3–4.4)	0.7 (0.5–0.9)	2.1 (1.7–2.5)	15.0 (14.0–16.0)	2.3 (1.9–2.7)
White, non-Hispanic	96,908	16.9 (16.4–17.5)	6.3 (6.0–6.7)	4.2 (3.9–4.5)	4.2 (3.9–4.6)	24.8 (24.1–25.4)***	5.5 (5.2–5.9)***
Black, non-Hispanic	17,131	14.9 (13.8–16.0)	7.0 (6.2–7.8)	0.8 (0.6–1.0)	2.2 (1.8–2.7)	20.6 (19.3–21.9)	3.7 (3.1–4.2)
Other	10,111	11.8 (10.6–13.1)	3.0 (2.3–3.6)	1.2 (0.7–1.7)	3.4 (2.6–4.3)	15.7 (14.3–17.1)	3.1 (2.4–4.2)
**Education**							
≤High school, GED	45,932	23.6 (22.8–24.4)	5.4 (4.9–5.8)	4.3 (3.8–4.7)	4.6 (4.1–5.0)	30.1 (29.2–31.0)***	6.2 (5.7–6.7)***
>High school	101,999	11.7 (11.2–12.2)	6.0 (5.6–6.4)	2.5 (2.2–2.7)	3.2 (2.9–3.4)	18.4 (17.8–19.0)	3.9 (3.6–4.2)
Unknown	550	—^†††^	—^†††^	—^†††^	—^†††^	—^†††^	—^†††^
**Poverty index^§§§^**							
Poor	11,313	22.9 (21.4–24.4)	6.3 (5.4–7.2)	2.3 (1.7–2.9)	4.4 (3.7–5.1)	28.5 (26.9–30.2)***	6.1 (5.3–6.9)***
Near poor	21,065	22.9 (21.7–24.0)	5.2 (4.6–5.9)	2.6 (2.1–3.0)	5.1 (4.4–5.8)	28.1 (26.8–29.4)***	6.2 (5.5–6.9)***
Not poor	107,453	13.4 (12.9–13.9)	6.0 (5.6–6.4)	3.2 (3.0–3.6)	3.3 (3.0–3.6)	20.6 (19.9–21.2)	4.3 (4.0–4.6)
Unknown	8,650	12.1 (10.6–13.7)	3.6 (2.7–4.6)	2.2 (1.5–2.9)	2.9 (2.0–3.7)	17.1 (15.2–19.0)	2.8 (2.1–3.5)
**Health insurance**							
Not insured	17,095	27.5 (26.1–28.9)	7.0 (6.2–7.8)	3.4 (2.8–4.0)	5.5 (4.7–6.3)	33.9 (32.3–35.5)***	7.7 (6.9–8.5)***
Insured	130,460	13.8 (13.4–14.2)	5.6 (5.3–5.9)	3.0 (2.7–3.2)	3.3 (3.1–3.5)	20.5 (20.0–21.0)	4.2 (3.9–4.5)
Unknown	926	—^†††^	—^†††^	—^†††^	—^†††^	—^†††^	—^†††^
**U.S. Census region^¶¶¶^**							
Northeast	25,712	14.1 (13.2–15.1)	5.6 (4.8–6.4)	1.4 (1.1–1.8)	2.5 (2.0–3.0)	19.9 (18.7–21.1)	3.3 (2.7–3.8)
Midwest	34,657	18.8 (17.9–19.8)	5.9 (5.4–6.5)	4.1 (3.6–4.7)	3.9 (3.4–4.4)	25.8 (24.8–26.9)***	5.5 (5.0–6.0)
South	53,050	16.0 (15.3–16.7)	5.8 (5.3–6.3)	3.6 (3.2–4.0)	3.8 (3.3–4.2)	22.9 (22.0–23.8)	4.9 (4.5–5.4)
West	35,062	12.1 (11.4–12.8)	5.8 (5.2–6.4	2.3 (2.0–2.7)	3.9 (3.4–4.3)	18.7 (17.8–19.6)	4.3 (3.7–4.8)

**Abbreviations:** CI = confidence interval; GED = General Educational Development certificate or diploma.

* Adults who reported “working at a job or business”; “with a job or business but not at work”; or “working, but not for pay, at a family-owned job or business” during the week before the interview.

^†^ Weighted to provide national annual average estimates for current employment.

^§^ Cigarette smokers were defined as persons who reported smoking ≥100 cigarettes during their lifetime and who currently smoke every day or some days (estimated n = 22.8 million).

^¶^ Other combustible tobacco product users were defined as persons who reported smoking cigars, cigarillos, or little filtered cigars or smoking tobacco in a regular pipe, water pipe, or hookah at least once during their lifetime and who currently use every day or some days (estimated n = 8.4 million).

** Smokeless tobacco product users were defined as persons who reported using chewing tobacco, snuff, dip, snus, or dissolvable tobacco at least once during their lifetime and who currently use every day or some days (estimated n = 4.4 million).

^††^ E-cigarette users were defined as persons reported who reported using electronic cigarettes at least once during their lifetime and who currently use every day or some days (n = 5.2 million).

^§§^ Any tobacco product users were defined as persons who reported current use of cigarettes or other combustible tobacco or smokeless tobacco or e-cigarettes every day or some days (estimated n = 32.7 million).

^¶¶^ Persons who reported current use of two or more individual tobacco products (estimated n = 6.9 million).

*** Statistically significant differences (p<0.05).

^†††^ Estimate suppressed (relative standard error >30%).

^§§§^ Poverty status is based on family income and family size using the U.S. Census Bureau's poverty thresholds for the previous calendar year. In National Health Interview Survey, “poor” persons are defined as having incomes below the poverty threshold, “near poor” are defined as having incomes of 100% to less than 200% of the poverty threshold, and “not poor” are defined as having incomes that are 200% of the poverty threshold or greater. Additional information available at ftp.cdc.gov/pub/Health_Statistics/NCHS/Dataset_Documentation/NHIS/2015/srvydesc.pdf.

^¶¶¶^ https://www2.census.gov/geo/pdfs/maps-data/maps/reference/us_regdiv.pdf.

Current tobacco use varied by industry (Table 2) and occupation (Table 3). Workers in the construction industry (34.3%) and installation, maintenance, and repair occupations (37.2%) had the highest reported use of any tobacco. Multiple tobacco product use was highest among workers in the construction industry (7.1%) and installation, maintenance, and repair occupations (10.1%). Cigarette smoking was highest among workers in the accommodation and food services industry (24.0%) and construction and extraction occupations (25.8%). Other combustible tobacco product use was highest among workers in the utilities industry (9.0%) and protective services occupations (10.2%). Smokeless tobacco use was highest among workers in the mining industry (14.3%) and installation, maintenance and repair occupations (9.6%). E-cigarette use was highest among workers in the accommodation and food services industry (5.8%) and installation, maintenance, and repair occupations (7.9%).

**TABLE 2:** Estimated prevalence of current tobacco use among working* adults, by tobacco product type and industry — National Health Interview Survey, United States, 2014–2016

Industry group	No. currently employed adults^†^ (x 1000)	% (95% CI)					
		Cigarette smokers^§^	Other combustible tobacco products^¶^	Smokeless tobacco products**	E-cigarettes^††^	Any tobacco product^§§^	≥2 Tobacco products^¶¶^
Accommodation and Food Services	9,907	24.0 (22.2–25.7)	6.9 (5.6–8.1)	2.1 (1.4–2.8)	5.8 (4.7–6.8)	29.9 (28.0–31.9)	7.0 (5.9–8.1)
Construction	9,346	23.4 (21.6–25.3)	7.9 (6.7–9.1)	7.8 (6.5–9.0)	4.2 (3.3–5.1)	34.3 (32.3–36.3)	7.1 (6.0–8.3)
Administrative and Support and Waste Management and Remediation Services	6,641	22.4 (20.3–24.5)	6.9 (5.5–8.3)	3.8 (2.7–5.0)	5.2 (3.9–6.4)	30.0 (27.8–32.3)	6.9 (5.4–8.4)
Transportation and Warehousing	6,052	20.3 (18.2–22.3)	7.4 (5.9–8.9)	5.3 (4.0–6.5)	5.2 (3.6–6.7)	30.2 (27.6–32.8)	6.5 (5.1–7.9)
Manufacturing	14,940	19.6 (18.2–20.9)	6.6 (5.4–7.8)	4.9 (4.2–5.6)	3.9 (2.8–5.1)	27.3 (25.7–28.9)	5.9 (4.9–7.0)
Retail Trade	14,968	17.8 (16.5–19.1)	6.1 (5.3–6.9)	2.3 (1.8–2.9)	4.8 (4.1–5.6)	24.3 (22.9–25.8)	5.5 (4.7–6.4)
Mining	859	17.5 (10.6–24.4)	5.2 (2.7–7.7)	14.3 (6.7–21.8)	—***	30.4 (23.3–37.5)	—***
Other Services (except Public Administration)	7,346	16.1 (14.3–17.9)	5.6 (4.3–6.8)	2.1 (1.5–2.8)	4.2 (3.1–5.2)	21.2 (19.1–23.2)	5.5 (4.3–6.7)
Wholesale Trade	3,810	16.0 (13.4–18.7)	6.5 (4.7–8.4)	4.0 (2.6–5.4)	3.6 (2.4–4.8)	24.2 (21.2–27.2)	4.9 (3.5–6.4)
Real Estate and Rental and Leasing	2,932	14.9 (12.3–17.5)	5.5 (3.8–7.2)	2.8 (1.5–4.1)	3.6 (2.2–5.0)	21.9 (18.8–25.0)	4.2 (2.7–5.6)
Agriculture, Forestry, Fishing, and Hunting	2,105	14.3 (11.5–17.2)	3.9 (2.5–5.3)	7.3 (5.2–9.5)	—***	21.4 (18.0–24.8)	5.0 (3.3–6.8)
Utilities	1,350	13.4 (9.4–17.4)	9.0 (5.7–12.4)	8.8 (4.5–13.1)	—***	25.3 (19.6–31.1)	5.4 (3.1–7.8)
Health Care and Social Assistance	19,755	13.0 (11.9–14.1)	3.3 (2.8–3.9)	1.2 (0.8–1.5)	2.4 (1.9–2.8)	16.4 (15.1–17.7)	2.7 (2.3–3.2)
Information	3,071	11.7 (9.3–14.0)	6.6 (4.8–8.5)	1.9 (0.9–2.9)	3.2 (1.8–4.5)	19.3 (16.4–22.2)	3.2 (2.0–4.5)
Finance and Insurance	6,775	11.2 (9.5–12.8)	5.6 (4.2–6.9)	1.7 (0.8–2.6)	3.2 (2.2–4.1)	17.6 (15.6–19.7)	3.2 (2.2–4.3)
Arts, Entertainment, and Recreation	3,059	11.1 (9.1–13.0)	6.4 (4.5–8.3)	2.3 (1.0–3.5)	3.6 (2.2–4.9)	17.4 (14.9–19.9)	5.1 (3.5–6.8)
Public Administration	7,358	10.9 (9.5–12.3)	6.4 (5.1–7.7)	3.8 (2.9–4.8)	2.1 (1.5–2.7)	19.0 (17.1–20.9)	3.6 (2.7–4.4)
Professional, Scientific, and Technical Services	11,286	9.6 (8.4–10.8)	7.1 (6.1–8.2)	1.5 (1.1–1.9)	3.9 (3.1–4.7)	17.7 (16.2–19.2)	3.4 (2.7–4.1)
Education services	14,135	7.2 (6.3–8.0)	3.3 (2.7–4.0)	1.2 (0.8–1.6)	1.4 (1.1–1.8)	11.0 (10.0–12.1)	1.7 (1.3–2.1)
Armed Forces	224	—***	—***	—***	—***	—***	—***
Management of Companies and Enterprises	83	—***	—***	—***	—***	—***	—***

**Abbreviation:** CI = confidence interval.

* Adults who reported “working at a job or business”; “with a job or business but not at work”; or “working, but not for pay, at a family-owned job or business” during the week before the interview.

^†^ Weighted to provide national annual average estimates for current employment.

^§^ Cigarette smokers were defined as persons who reported smoking ≥100 cigarettes during their lifetime and who currently smoke every day or some days (estimated n = 22.8 million).

^¶^ Other combustible tobacco product users were defined as persons who reported smoking cigars, cigarillos, or little filtered cigars or smoking tobacco in a regular pipe, water pipe, or hookah at least once during their lifetime and who currently use every day or some days (estimated n = 8.4 million).

** Smokeless tobacco product users were defined as persons who reported using chewing tobacco, snuff, dip, snus, or dissolvable tobacco at least once during their lifetime and who currently use every day or some days (estimated n = 4.4 million).

^††^ E-cigarette users were defined as persons who reported using electronic cigarettes at least once during their lifetime and who currently use every day or some days (n = 5.2 million).

^§§^ Any tobacco product users were defined as persons who reported current use of cigarettes or other combustible tobacco or smokeless tobacco or e-cigarettes every day or some days (estimated n = 32.7 million).

^¶¶^ Persons who reported current use of two or more individual tobacco products (estimated n = 6.9 million).

*** Estimate suppressed (relative standard error >30%).

**TABLE 3:** Estimated prevalence of current tobacco use among working* adults, by tobacco product type and occupation — National Health Interview Survey, United States, 2014–2016

Occupation group	No. currently employed adults^†^ (x 1000)	% (95% CI)					
		Cigarette smokers^§^	Other combustible tobacco products^¶^	Smokeless tobacco products**	E-cigarettes^††^	Any tobacco product^§§^	≥2 Tobacco products^¶¶^
Construction and Extraction	7,175	25.8 (23.7–28.0)	7.3 (5.9–8.7)	9.0 (7.5–10.4)	3.9 (3.0–4.8)	36.5 (34.1–38.9)	7.5 (6.2–8.9)
Food Preparation and Serving Related	7,501	25.1 (22.9–27.3)	6.5 (5.1–7.8)	1.7 (1.1–2.4)	5.3 (4.2–6.4)	29.8 (27.5–32.1)	6.8 (5.6–8.0)
Production	8,563	23.7 (21.8–25.6)	6.7 (5.6–7.8)	5.8 (4.8–6.7)	4.2 (3.3–5.1)	31.1 (29.0–33.3)	7.4 (6.3–8.5)
Installation, Maintenance, and Repair	5,043	23.1 (19.6–26.5)	10.1 (7.2–12.9)	9.6 (7.5–11.7)	7.9 (5.2–10.7)	37.2 (33.0–41.3)	10.1 (6.7–13.4)
Transportation and Material Moving	8,410	22.5 (20.6–24.4)	7.7 (6.4–8.9)	5.2 (4.4–6.1)	5.1 (4.0–6.2)	31.8 (29.7–33.9)	7.0 (5.8–8.2)
Building and Grounds Cleaning and Maintenance	5,896	22.0 (19.7–24.3)	4.8 (3.5–6.0)	2.9 (1.9–3.9)	3.3 (2.4–4.2)	26.5 (24.0–29.0)	5.3 (4.0–6.5)
Healthcare Support	3,298	18.6 (15.7–21.5)	2.4 (1.4–3.5)	1.3 (0.6–2.0)	3.4 (2.3–4.6)	21.8 (18.7–24.8)	3.3 (2.2–4.5)
Personal Care and Service	5,281	17.6 (14.2–21.0)	4.9 (3.6–6.2)	—***	4.0 (2.9–5.2)	21.4 (17.9–24.9)	5.2 (3.9–6.5)
Office and Administrative Support	17,481	16.3 (15.2–17.4)	3.8 (3.2–4.4)	1.2 (0.9–1.6)	4.1 (3.4–4.9)	21.1 (19.8–22.3)	3.9 (3.3–4.4)
Protective Service	3,067	15.8 (12.8–18.7)	10.2 (7.7–12.6)	8.3 (6.1–10.6)	3.5 (2.0–5.0)	29.1 (25.5–32.7)	6.8 (4.4–9.1)
Farming, Fishing, and Forestry	1,128	15.6 (11.7–19.5)	4.2 (2.4–6.1)	8.9 (5.8–12.1)	—***	23.8 (19.3–28.3)	5.6 (3.2–8.0)
Sales and Related	14,639	15.2 (13.9–16.5)	6.9 (6.0–7.9)	2.7 (2.0–3.4)	4.2 (3.5–4.9)	22.7 (21.2–24.2)	5.0 (4.2–5.8)
Management	14,856	12.0 (10.9–13.1)	6.9 (6.0–7.7)	3.0 (2.4–3.6)	3.0 (2.4–3.6)	19.8 (18.4–21.2)	4.0 (3.3–4.6)
Computer and Mathematical	5,218	9.6 (7.9–11.2)	5.8 (4.7–7.0)	1.3 (0.7–2.0)	2.8 (1.9–3.7)	16.5 (14.4–18.5)	2.6 (1.8–3.3)
Business and Financial Operations	7,664	9.2 (7.9–10.5)	5.3 (4.2–6.4)	1.9 (1.1–2.7)	2.5 (1.8–3.2)	15.0 (13.4–16.7)	3.1 (2.3–3.9)
Community and Social Services	2,756	8.9 (6.8–11.0)	5.3 (3.3–7.2)	—***	2.2 (1.3–3.1)	13.5 (11.0–16.1)	2.7 (1.3–4.0)
Architecture and Engineering	3,295	8.8 (6.7–10.8)	7.8 (5.6–10.0)	2.9 (1.7–4.2)	3.0 (1.7–4.4)	18.3 (15.2–21.3)	3.7 (2.3–5.1)
Arts, Design, Entertainment, Sports, and Media	3,083	8.7 (6.9–10.6)	7.2 (5.4–9.1)	1.8 (0.9–2.8)	2.9 (1.5–4.2)	16.7 (14.1–19.2)	3.2 (1.9–4.5)
Healthcare Practitioners and Technical	8,642	8.1 (6.9–9.3)	2.8 (2.1–3.6)	0.9 (0.4–1.3)	2.2 (1.5–2.8)	11.7 (10.4–13.1)	2.0 (1.3–2.6)
Legal	1,766	7.3 (5.0–9.5)	5.7 (3.5–8.0)	—***	2.4 (1.0–3.7)	14.1 (11.1–17.1)	—***
Education, Training, and Library	9,474	5.7 (4.7–6.6)	3.3 (2.5–4.1)	1.2 (0.6–1.7)	1.3 (0.9–1.8)	9.5 (8.3–10.8)	1.5 (1.0–2.0)
Life, Physical, and Social Science	1,535	5.6 (3.5–7.7)	3.9 (2.1–5.7)	—***	—***	9.3 (6.8–11.8)	—***
Military	234	—***	—***	—***	—***	—***	—***

**Abbreviation:** CI = confidence interval.

* Adults who reported “working at a job or business”; “with a job or business but not at work”; or “working, but not for pay, at a family-owned job or business” during the week before the interview.

^†^ Weighted to provide national annual average estimates for current employment.

^§^ Cigarette smokers were defined as persons who reported smoking ≥100 cigarettes during their lifetimes and who currently smoke every day or some days (estimated n = 22.8 million).

^¶^ Other combustible tobacco product users were defined as persons who reported smoking cigars, cigarillos, or little filtered cigars or smoking tobacco in a regular pipe, water pipe, or hookah at least once during their lifetime and who currently use every day or some days (estimated n = 8.4 million).

** Smokeless tobacco product users were defined as persons who reported using chewing tobacco, snuff, dip, snus, or dissolvable tobacco at least once during their lifetime and who currently use every day or some days (estimated n = 4.4 million).

^††^ E-cigarettes users were defined as persons who reported using electronic cigarettes at least once during their lifetime and who currently use every day or some days (n = 5.2 million).

^§§^ Any tobacco product users were defined as persons who reported current use of cigarettes or other combustible tobacco or smokeless tobacco or e-cigarettes every day or some days (estimated n = 32.7 million).

^¶¶^ Persons who reported current use of two or more individual tobacco products (estimated n = 6.9 million).

*** Estimate suppressed (relative standard error >30%).

## Discussion

During 2014–2016, an estimated one in five working U.S. adults (32.7 million; 22.1%) currently used some form of tobacco, and cigarettes were the most commonly used tobacco product. Overall, a decline in cigarette smoking, smokeless tobacco, and e-cigarette use was observed among U.S. workers (*2*,*4*). However, tobacco use varied by product type, sociodemographic characteristics, and industry and occupation, with a higher prevalence of any tobacco use among workers in the construction industries and installation, maintenance, and repair occupations. These findings underscore the importance of implementation of evidence-based interventions, in coordination with continued surveillance of all forms of tobacco products use, to reduce tobacco-related disease and death** among U.S. working adults, particularly industry and occupation groups with higher tobacco use prevalences (*1*).

Among working adult tobacco users, an estimated 6.9 million adults used two or more tobacco products. Use of multiple tobacco products is associated with increased risk for nicotine addiction, dependence, and adverse health effects (*3*,*8*). These health effects can lead to increased risks for tobacco-related morbidity and mortality (*3*). In addition, variations in multiple tobacco product use were observed across population groups, which is consistent with previous findings of higher prevalences of combustible and smokeless tobacco use among workers in certain industries and occupations (*2*). These findings underscore the importance of opportunities for targeted efforts to reduce tobacco use among populations with the greatest prevalence of tobacco use, including multiple tobacco product users.

The findings in this report are subject to at least three limitations. First, the collected employment information applied only to the week before the interview. Some workers might have changed jobs, and thus, might have been in a different occupation or industry at the time of the survey interview. However, supplemental analyses examining the longest held job yielded similar results. Second, the extent of under- or overreporting of tobacco use could not be determined because tobacco use information was self-reported, and thus, was not validated by biochemical tests. However, comparison of self-reported smoking status with measured serum cotinine levels suggests generally high levels of correlation (*9*). Finally, estimates for some groups (e.g., management of companies and enterprises industry workers) and tobacco product use were unreliable and suppressed because of small sample sizes.

Continued implementation of proven strategies to address tobacco use among U.S. adults is important (*6*,*8*,*10*). Proven strategies include anti-tobacco messages; comprehensive tobacco-free laws covering public places and worksites; providing comprehensive coverage for tobacco cessation treatments for employees; increased tobacco prices; and tailored interventions that help prevent initiation and encourage cessation among workers. Workplace tobacco-control interventions have been especially effective in reducing cigarette smoking prevalence (*7*). Previous research has indicated that workers at worksites that adopted or maintained smoke-free policies were twice as likely to quit smoking than those whose worksites did not implement such policies (*7*). To maximize the health of workers, employers can also consider integrating comprehensive and effective tobacco cessation programs into workplace health promotion programs (*7*,*10*).

**Summary**

**What is already known about this topic?**

Differences exist in tobacco use by industry and occupation among U.S. working adults. Workplace tobacco-control interventions have been effective in reducing cigarette smoking prevalence and exposure to secondhand smoke.

**What is added by this report?**

Analysis of National Health Interview Survey data for 2014–2016 found that among working adults, 22.1% currently (every day or some days) used any form of tobacco product; 15.4% currently used cigarettes, 5.8% used other combustible tobacco products, 3.0% used smokeless tobacco, and 3.6% used electronic cigarettes; overall, 4.6% used two or more tobacco products. By industry, any tobacco product use ranged from 11.0% among education services to 34.3% among construction workers; use of two or more tobacco products was highest among construction industry workers. By occupation, any tobacco use ranged from 9.3% among life, physical, and social science workers to 37.2% among installation, maintenance, and repair workers; use of two or more tobacco products was highest among installation, maintenance, and repair workers.

**What are the implications for public health action?**

These findings underscore the importance of continued implementation of proven strategies to prevent and reduce tobacco product use, including current use of multiple products among working adults. To maximize the health of workers, employers could also consider integrating comprehensive and effective tobacco cessation programs into health promotion programs in the workplace.

## Footnotes

* https://ftp.cdc.gov/pub/Health_Statistics/NCHS/Dataset_Documentation/NHIS/2015/srvydesc.pdf.

† Additional information about industry and occupation groups and codes is available at ftp://ftp.cdc.gov/pub/Health_Statistics/NCHS/Dataset_Documentation/NHIS/2015/samadult_layout.pdf on pages 378–384.

§ https://www.cdc.gov/nchs/data/series/sr_10/sr10_256.pdf.

¶ Poverty status is based on family income and family size using the U.S. Census Bureau’s poverty thresholds for the previous calendar year. In the National Health Interview Survey, “'poor” persons are defined as having incomes less than the poverty threshold, “near poor”' are defined as having incomes of 100% to less than 200% of the poverty threshold, and “not poor” are defined as having incomes that are 200% of the poverty threshold or greater. ftp://ftp.cdc.gov/pub/Health_Statistics/NCHS/Dataset_Documentation/NHIS/2015/samadult_layout.pdf.

** Task Force on Community Preventive Services. https://www.thecommunityguide.org/tobacco/tobacco.pdf.
